# Ikbkap/Elp1 Deficiency Causes Male Infertility by Disrupting Meiotic Progression

**DOI:** 10.1371/journal.pgen.1003516

**Published:** 2013-05-23

**Authors:** Fu-Jung Lin, Li Shen, Chuan-Wei Jang, Pål Ø. Falnes, Yi Zhang

**Affiliations:** 1 Howard Hughes Medical Institute, Harvard Medical School, Boston, Massachusetts, United States of America; 2 Program in Cellular and Molecular Medicine, Boston Children's Hospital, Harvard Medical School, Boston, Massachusetts, United States of America; 3 Department of Genetics, Harvard Medical School, Boston, Massachusetts, United States of America; 4 Department of Biosciences, University of Oslo, Oslo, Norway; 5 Harvard Stem Cell Institute, Harvard Medical School, Boston, Massachusetts, United States of America; Cornell University, United States of America

## Abstract

Mouse *Ikbkap* gene encodes IKAP—one of the core subunits of Elongator—and is thought to be involved in transcription. However, the biological function of IKAP, particularly within the context of an animal model, remains poorly characterized. We used a loss-of-function approach in mice to demonstrate that *Ikbkap* is essential for meiosis during spermatogenesis. Absence of *Ikbkap* results in defects in synapsis and meiotic recombination, both of which result in increased apoptosis and complete arrest of gametogenesis. In *Ikbkap-*mutant testes, a few meiotic genes are down-regulated, suggesting IKAP's role in transcriptional regulation. In addition, *Ikbkap*-mutant testes exhibit defects in wobble uridine tRNA modification, supporting a conserved tRNA modification function from yeast to mammals. Thus, our study not only reveals a novel function of IKAP in meiosis, but also suggests that IKAP contributes to this process partly by exerting its effect on transcription and tRNA modification.

## Author Summary

The process of meiosis is responsible for gamete formation and ensures that offspring will inherit a complete set of chromosomes from each parent. Errors arising during this process generally result in spontaneous abortions, birth defects, or infertility. Many genes that are essential in regulating meiosis have also been implicated in DNA repair. Importantly, defects in DNA repair are common causes of cancers. Therefore, identification of genes important for normal meiosis contributes not only to the field of reproduction but also to the field of cancer biology. We studied the effects of deleting mouse *Ikbkap*, a gene that encodes one of the subunit of the Elongator complex initially described as an RNA polymerase II–associated transcription elongation factor. We demonstrate that *Ikbkap* mutant mice exhibit infertility and defects in meiotic progression. Specifically, homologous and sex chromosomes fail to synapse (become associated), DNA double-strand breaks are inefficiently repaired, and DNA crossovers are significantly decreased in *Ikbkap* males. We also demonstrate that the requirement for Elongator in tRNA modification, which has been shown in lower eukaryotes, is conserved in mammals. Our findings suggest novel roles for *Ikbkap* in meiosis progression and tRNA modification, which have not been reported previously.

## Introduction

Meiosis is a fundamental and highly regulated process that takes place during gamete generation. Faithful execution of this process is essential for maintaining genome integrity. Errors and various types of disruption during meiosis can cause aneuploidy and result in developmental defects, including mental retardation in Trisomy 21, infertility, to name two [1].

During the prophase I stage of the first meiotic division, homologous chromosomes undergo pairing and synapsis. Synapsis is mediated by a protein complex namely the synaptonemal complex (SC), and is accompanied by chromosome recombination [2]. Unlike homologous autosomes, the X and Y chromosome synapsis occurs only at a very small region of homology, a pseudoautosomal region (PAR) [3]. Formation of the fully synapsed autosomal SCs as well as the partially synapsed sex chromosome are essential for DNA repair, recombination and subsequent desynapsis [4]. Consequently, DNA damage response (DDR) is initiated upon the recognition of the DNA lesion made by SPO11, which is a type II-like topoisomerase that induces double-stranded breaks (DSBs) [5]. At the DSB sites, the DNA repair machinery generates DNA recombination between homologous chromosomes to ensure proper disjunction at metaphase I. The genetic studies in yeast and mouse helped identify many factors important for meiosis [6], [7], [8], such as: the master regulators meiosis-inducing protein 1 (Ime1) in yeast, and A-MYB (MYBL1) in mouse [9], [10]. Despite great progress in understanding the transcriptional regulation of the meiotic process [2], very little is known about the role of translational control during this process. Our data presents evidence that the evolutionarily conserved factor *Ikbkap/Elp1* governs meiotic progression at the level of both transcription and translation.

Elp1, also referred to as IKAP (Inhibitor of kappaB kinase -associated protein), functions as a scaffold protein that assembles the Elongator and is encoded by *Ikbkap* gene (we will use the MGI nomenclature, IKAP for the protein, and *Ikbkap* for the gene, hereafter). Elongator is a protein complex comprised of two copies of the core complex, Elp1–3, and a sub-complex, Elp4–6 [11]. The protein complex “Elongator” was first purified in budding yeast through its association with the elongating RNA polymerase II (RNAP II) [12]. Similar protein complex was subsequently purified from human cells [13], [14], [15]. Interestingly, the components of the protein complex are highly conserved in different species that include yeast and human. The Elongator complex has important biological functions as deletion or mutation of any of its subunits results in severe phenotypes in yeast. Among the Elongator components, Elp3 likely serves as a catalytic subunit, because it not only harbors motifs characteristic of the GCN5 family of histone acetyltransferases (HATs), but also has been shown to directly acetylate H3 lysine 14 (H3K14) and possibly H4K8 *in vitro* [16]. These findings, combined with the studies demonstrating the association of Elongator with RNAP II holoenzyme, its ability to bind to nascent pre-mRNA, and to facilitate RNAPII transcribes through chromatin in an acetyl-CoA-dependent manner, support its role in transcription regulation [12], [14], [17].

Accumulating evidence suggest that Elongator, in addition to participating in transcriptional regulation, also plays pivotal role in the regulation of translation. The first evidence implicating the involvement of the Elongator in translation came from a genetic screen, which demonstrated that all genes encoding the yeast Elongator subunits are required for the formation of 5-carbamoylmethyl (ncm^5^), and 5-methoxycarbonylmethyl (mcm^5^) side chains on uridines at the wobble position of certain tRNAs [18]. These modified nucleosides are important for efficient decoding of A- and G- ending codons through stabilizing codon-anticodon interactions during translation [19], [20], [21]. Studies have shown that all the six subunits of the Elongator are required for the early step of mcm and ncm side chain formation [18]. Although it is currently unclear how Elongator contributes to the generation of the modified tRNAs, this function is conserved in *S. cerevisiae*, *S. pombe*, *C. elegans*, and *A. thaliana* [18], [22], [23], [24]. Whether such function is conserved in mammals remains to be determined.

Using a loss-of-function approach, we demonstrate that IKAP plays an important role in male meiosis. First, we show that IKAP is highly expressed in male germ cells. Targeted deletion of *Ikbkap* in mice resulted in increased apoptosis in male germ cells and male infertility. Interestingly, autosomal and sex chromosome synapsis defects are observed in *Ikbkap* mutant spermatocytes. In addition, sustained RAD51 foci are observed on the autosomes of mutant spermatocytes, suggesting a homologous recombination repair defect. Detailed molecular studies revealed that the expression of a few meiotic genes is down-regulated in mutant testes. Furthermore, the levels of the Elongator-dependent tRNA modifications are reduced in the mutant testes. Our study thus reveals a critical function of *Ikbkap* in male meiosis, and demonstrates a conservation tRNA modification function in mammalian cells.

## Results

### IKAP expression is restricted to germ cells during spermatogenesis

**Figure 1:**
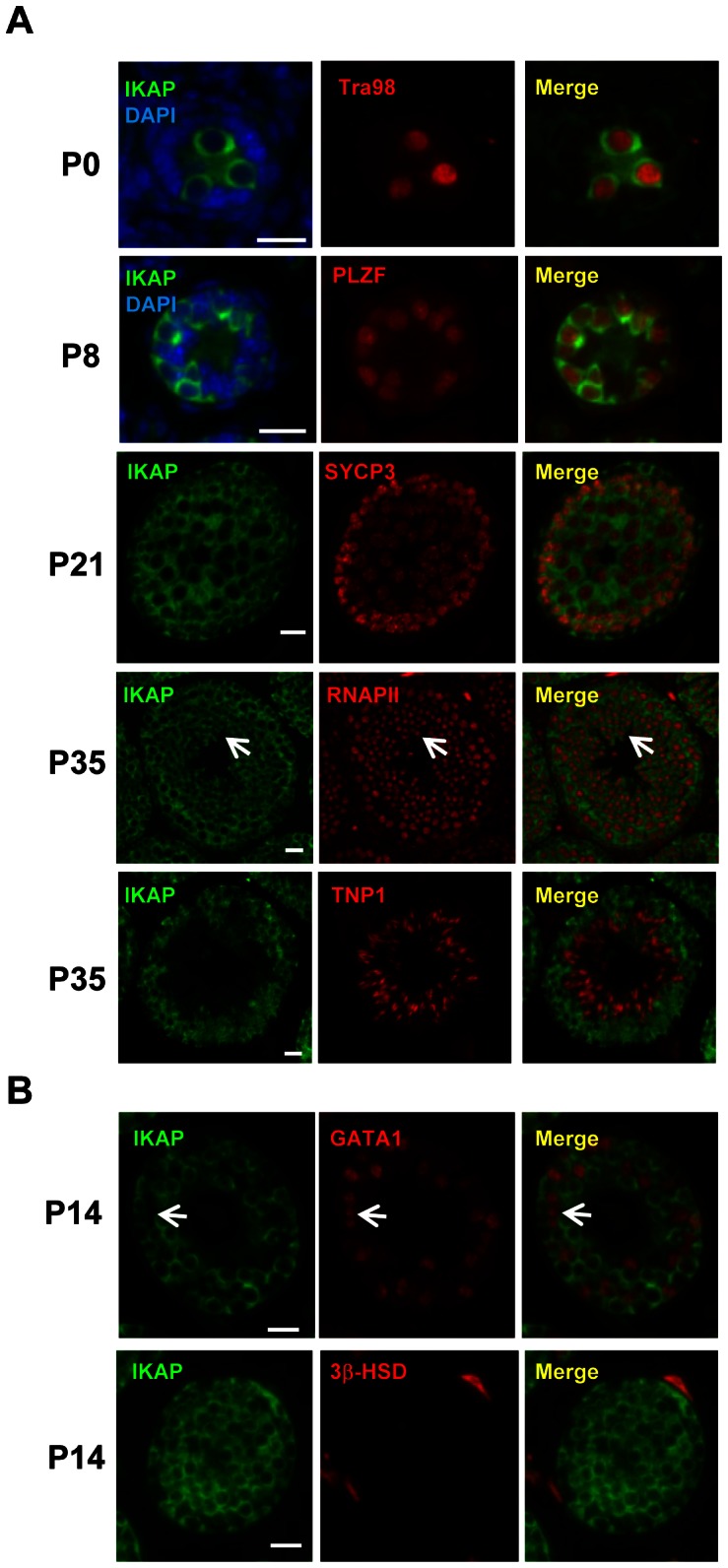
Germ cell-specific expression of IKAP during spermatogenesis. (A) IKAP is specifically detected in the gonocytes, and is colocalized with the germ cell marker Tra98 at P0, and the undifferentiated spermatogonia marker PLZF at P8. IKAP is detectable in the primary spermatocytes marked by SYCP3 at P21 and IKAP is expressed in the round spermatids (arrows) as indicated by RNAPII. However, IKAP is not detected in Tnp1-expressing elongated spermatids. Bar, 20 µm. (B) IKAP is not expressed in Sertoli cells or Leydig cells marked by GATA1 (arrows) or 3β-HSD, respectively. Bar, 20 µm.

To explore a possible role of IKAP in gametogenesis, we analyzed the expression pattern of IKAP during spermatogenesis by immunofluorescence staining. This analysis revealed that IKAP is expressed in Tra98-positive gonocytes as early as postnatal day 0 (P0) with a predominant cytoplasmic localization (Figure 1A). This expression and localization pattern is maintained at P8, as prospermatogonia developed into PLZF-positive undifferentiated spermatogonia (Figure 1A). At P21, IKAP expression remains in SYCP3-expressing meiocytes (Figure 1A). At late stage of spermatogenesis, IKAP was detected in RNA polymerase II-positive round spermatids (Figure 1A, arrows), but not in transition protein 1 (TNP1)-positive elongated spermatids at P35 (Figure 1A). In contrast to specific expression in germ cells, IKAP is undetectable in the GATA1-positive Sertoli cells (Figure 1B, arrow) or 3β-HSD positive Leydig cells (Figure 1B). We also used the conditional knockout testes (as below) as negative controls for the purpose of antibody validation (Figure S1). Collectively, immunofluorescence staining revealed that IKAP is expressed in all stages of male germ cells except the elongated spermatids, but it is almost undetectable in somatic cells of testis.

### *Ikbkap* deficiency in germ cells results in infertility

The germ cell-specific expression pattern of IKAP revealed above suggests that IKAP might have a role in spermatogenesis. Previous studies have demonstrated that *Ikbkap* null mutant mice die of cardiovascular and neuronal developmental defects at embryonic day E10 [25], [26]. To bypass the embryonic requirement for *Ikbkap*, we used mice harboring a conditional knockout allele for *Ikbkap* with exon 4 flanked by two *loxP* sites (Figure S2A). Mice homozygous for the *Ikbkap^flox^* conditional allele were viable and were born at Mendelian ratio (data not shown). To explore the function of *Ikbkap* in spermatogenesis, we inactivated *Ikbkap* in the male germ line by crossing with the *Vasa-Cre* mice (also known as *Ddx4-Cre*). *Vasa-Cre* induces recombination in germ cells starting from E15.5, and is expressed in all spermatogenic cells postnatally [27]. The germ linage conditional *Ikbkap* mutant mice (genotyped as *Vasa-Cre; Ikbkap^flox^* ^/−^, referred to as CKO hereafter) were obtained by crossing *Ikbkap^flox/flox^* females with *Vasa-Cre; Ikbkap^flox/+^* males. The genotypes of control mice were either *Vasa-Cre; Ikbkap^flox/+^*, or *Ikbkap^flox/−^*, or *Ikbkap^flox/+^*. RT-qPCR analysis using P16 mouse testes demonstrates that the deletion efficiency is more than 80% (Figure S2B). Western blot analysis and immunostaining using two commercial antibodies revealed marked reduction of IKAP protein in the CKO testes (Figure S2C, S2D and data not shown).

To test for a possible function of IKAP in spermatogenesis, CKO and control male mice were mated with wild-type females and the breeding capacity was monitored for 3 months. While the control mice gave birth at an average litter size 6.7±1.5, no pups were obtained from wild-type females mated with CKO males, even though copulatory plugs were frequently observed in the females. These results suggest that loss of function of *Ikbkap* in male mice causes infertility.

### Deletion of *Ikbkap* in germ cells causes spermatogenic arrest

**Figure 2:**
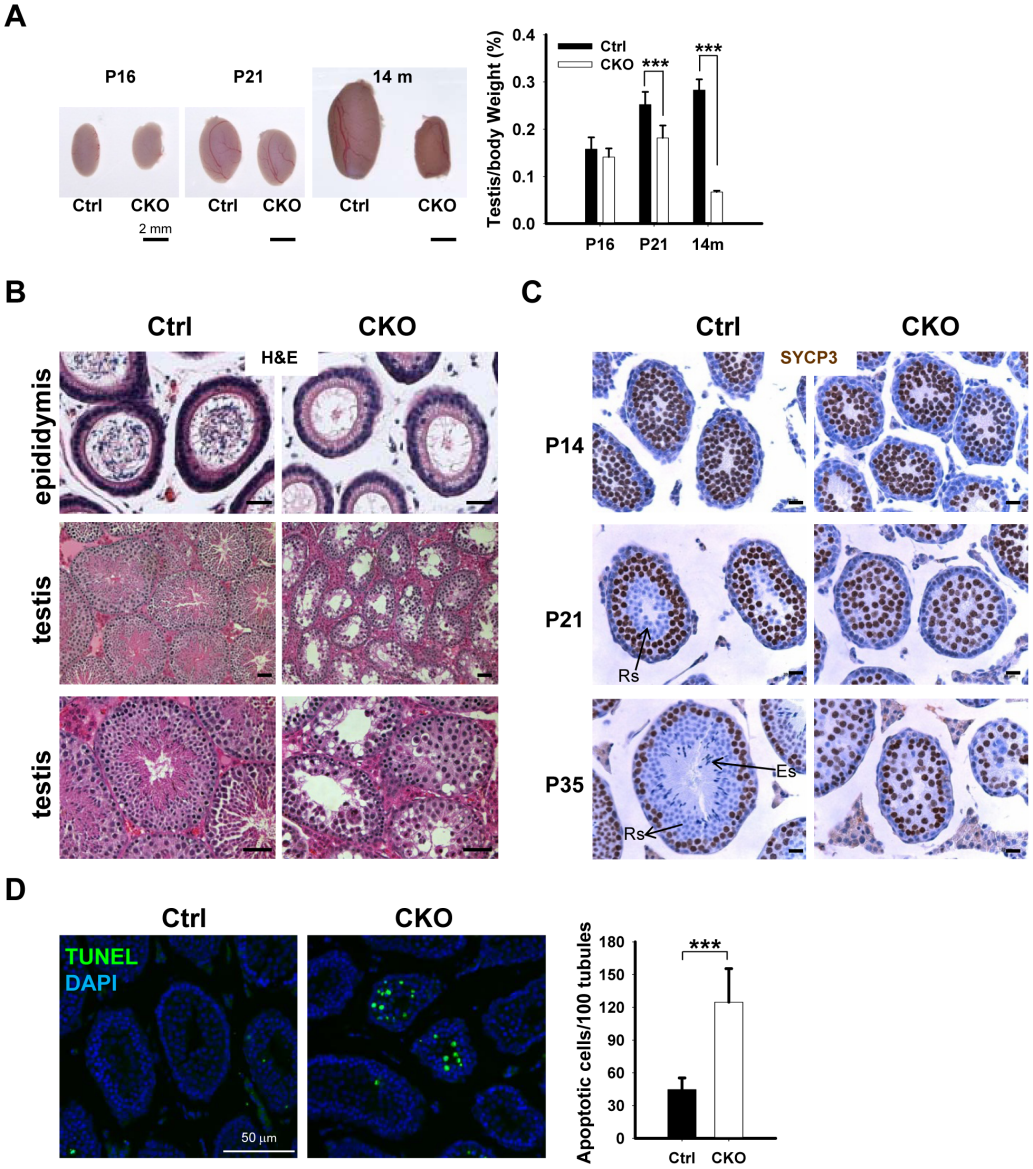
*Ikbkap* deletion results in germ cell loss in testes. (A) Left, representative image of whole testes of control and CKO mutants at P16, P21 and 14-mo-old. Right, quantification of the testes to body weight ratio. (***) *P*<0.001. At least 3 animals for each genotype were used for each stage. Bar, 2 mm. (B) Representative images of H&E stained paraffin sections from control and CKO mutant epididymis at 8-wk-old and from testes at10-wk-old. Bar, 200 µm. (C) SYCP3 staining (brown) of control and CKO mutant testes at P14, P21 and P35. Bar, 20 µm. ES, elongated spermatids; RS, round spermatids. (D) TUNEL assay and quantification of TUNEL positive cells were performed using P21 testes. There is a significant increase of TUNEL-positive cells in CKO testes relative to control testes. N = 3. Bar, 50 µm. (***) *P*<0.001.

To determine the potential cause of infertility, we examined the size of male gonads and the presence of spermatozoa in the epididymis. We found that the size of the testes is significantly decreased in the CKO mice, and the testicular weight to body weight ratio was reduced by 25% at the age of 14 month (Figure 2A). No spermatozoa were found in the epididymis of 2-month old CKO mice (Figure 2B). Histological analyses indicated stage IV (mid-pachytene) arrest of the seminiferous epithelium (Figure 2B), which is typical of diverse mouse meiotic mutants [28]. Indeed, in contrast to control seminiferous tubules, CKO testes lacked postmeiotic spermatids. To determine the stages at which *Ikbkap* deficiency causes germ cells perturbation, we examined the first round of spermatogenesis using juvenile testes. Immunostaining with the meiocyte maker SYCP3 revealed no obvious histological change in CKO testes at P14 (Figure 2C), suggesting that CKO germ cells enter meiosis and progress to the prophase I normally as in testes of control mice. However, at P21, while most of the tubules of control testes have postmeiotic round spermatids, CKO testes have almost no postmeiotic cells with SYCP3-expressing meiocytes disorganized and scattered through seminiferous tubules (Figure 2C). At P35, while control testes showed a full spectrum of spermatogenic cells, including primary spermatocytes, rounds spermatids, and spermatozoa, no post-meiotic cells, such as round and elongated spermatids, were found in the CKO testes (Figure 2C and Figure S3A). These results suggest that *Ikbkap* deletion causes spermatogenesis arrest at meiotic prophase (Figure 2C). Consistent with the lack of post-meiotic germ cells in the mutant testes, Terminal deoxynucleotidyl transferase dUTP nick end labeling (TUNEL) assays revealed an increase in the number of apoptotic cells in the CKO testes compared to that of the controls (Figure 2D; *p*<0.001), indicating that apoptosis at least partly explains the lack of round and elongated sperms in the CKO testes. Consistent with the absence of IKAP in somatic cells, no morphological change in Sertoli cells or Leydig cells was observed in CKO testes, and the density of GATA1-positive Sertoli cells and 3β-HSD-positive Leydig cells was also not altered in response to *Ikbkap* deletion (Figure S3B). Taken together, the above results demonstrate that *Ikbkap* deletion impedes spermatogenesis during meiotic stage, resulting in increased apoptosis.

### Deletion of *Ikbkap* affects meiotic progression

**Figure 3:**
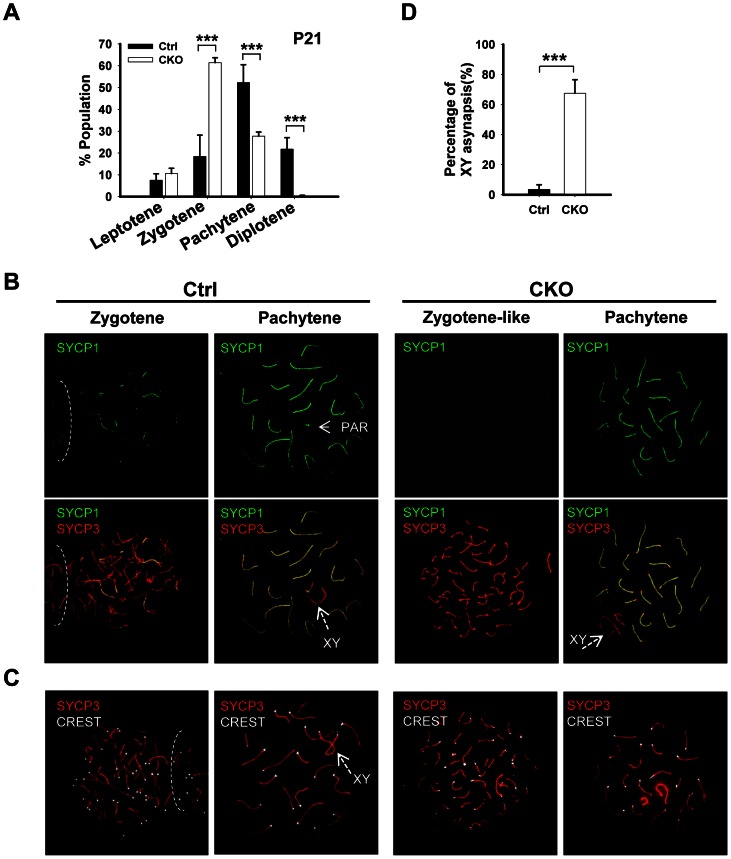
*Ikbkap* mutant spermatocytes exhibit synapsis defects. (A) Bar graph representation of the percentage of the spermatocytes in each meiotic prophase stages of the control and CKO mice. A total of around 170 cells per testis were counted. (***) *P*<0.001. (B) Representative images of chromosome spreads from control and CKO spermatocytes co-stained with SYCP1 (green) and SYCP3 (red) antibodies. (C) Representative images of chromosome spreads from control and CKO spermatocytes stained with SYCP3 (red) and CREST (white) antibodies. (D) The percentage of unsynapsed X and Y chromosomes in the pachytene spermatocytes. (***) *P*<0.001.

The lack of post-meiotic germ cells in the CKO testes prompted us to examine the impact of *Ikbkap* deletion on meiotic process. The meiotic prophase is divided into five stages, including leptotene, zygotene, pachytene, diplotene, and diakinesis [2]. We first examined the stage distribution of spermatocytes in control and CKO testes based on chromosomal morphology and sex body status. In the control P21 testes, pachytene spermatocytes are the most abundant, accounting for half of the total cell population, followed by diplotene, zygotene and leptotene stage cells (Figure 3A). In contrast, the CKO testes exhibited an accumulation of zygotene spermatocytes, and a significant decrease of pachytene spermatocytes (Figure 3A, *p*<0.01), suggesting that the impairment of meiotic progression takes place between zygotene to pachytene stage.

### *Ikbkap*-deficient spermatocytes exhibit aberrant synapsis

To characterize the observed meiotic defect in detail, we performed co-immunostaining of meiotic chromosome spreads using antibodies against axial/lateral (SYCP3) and central (SYCP1) elements of the synaptonemal complex. In normal meiosis, the axial element of synaptonemal complex starts to form at the leptotene stage (Figure S4). Synapsis is initiated at the zygotene stage, as determined by the appearance of SYCP1- a marker of fully synapsed chromosome segments (Figure 3B). Synapsis is completed at the pachytene stage as chromosome cores contained 20 fully synapsed bivalents, including XY pair synapsed at the pseudoautosomal region (PAR). Despite normal development of axial elements, CKO spermatocytes exhibited an increase in the unpaired SC at zygotene stage. Zygotene-like nuclei, with approximately 40 or more short fragmented stretches of SYCP3 and no SYCP1 staining were observed in 61.3% (n = 60) of CKO zygotene spermatocytes (Figure 3B), suggesting a synapsis defect. To further analyze the synaptic defects, we investigated the centromere distribution by immunostaining with centromere marker CREST and SYCP3. Prior to synapsis, 40 centromeres are usually observed in the control leptotene spermatocytes. As the synapsis progresses, the number of visible centromeres reduces and becomes 21 centromeric foci (19 from synapsed autosomes and 2 from the XY bivalent) at the pachytene stage (Figure 3C). In the CKO zygotene-like spermatocytes, we observed greater than 20 centromeric foci, most containing 40 CREST foci (Figure 3C), indicating CKO spermatocytes failed to complete homologous chromosome pairing. Although 27.7% of CKO spermatocytes proceed to pachytene stage and exhibit 19 fully synapsed autosomal bivalent chromosomes (Figure 3A and 3B), XY asynapsis, in which X and Y axes were not associated, were frequently observed in CKO spermatocytes (67.5%, n = 225), as judged by the absence of SYCP1 (Figure 3B and 3D). Taken together, *Ikbkap* deficiency in mouse spermatocytes leads to the disruption of synapsis.

### *Ikbkap* deficiency affects sex chromosome synapsis and DSB repair

**Figure 4:**
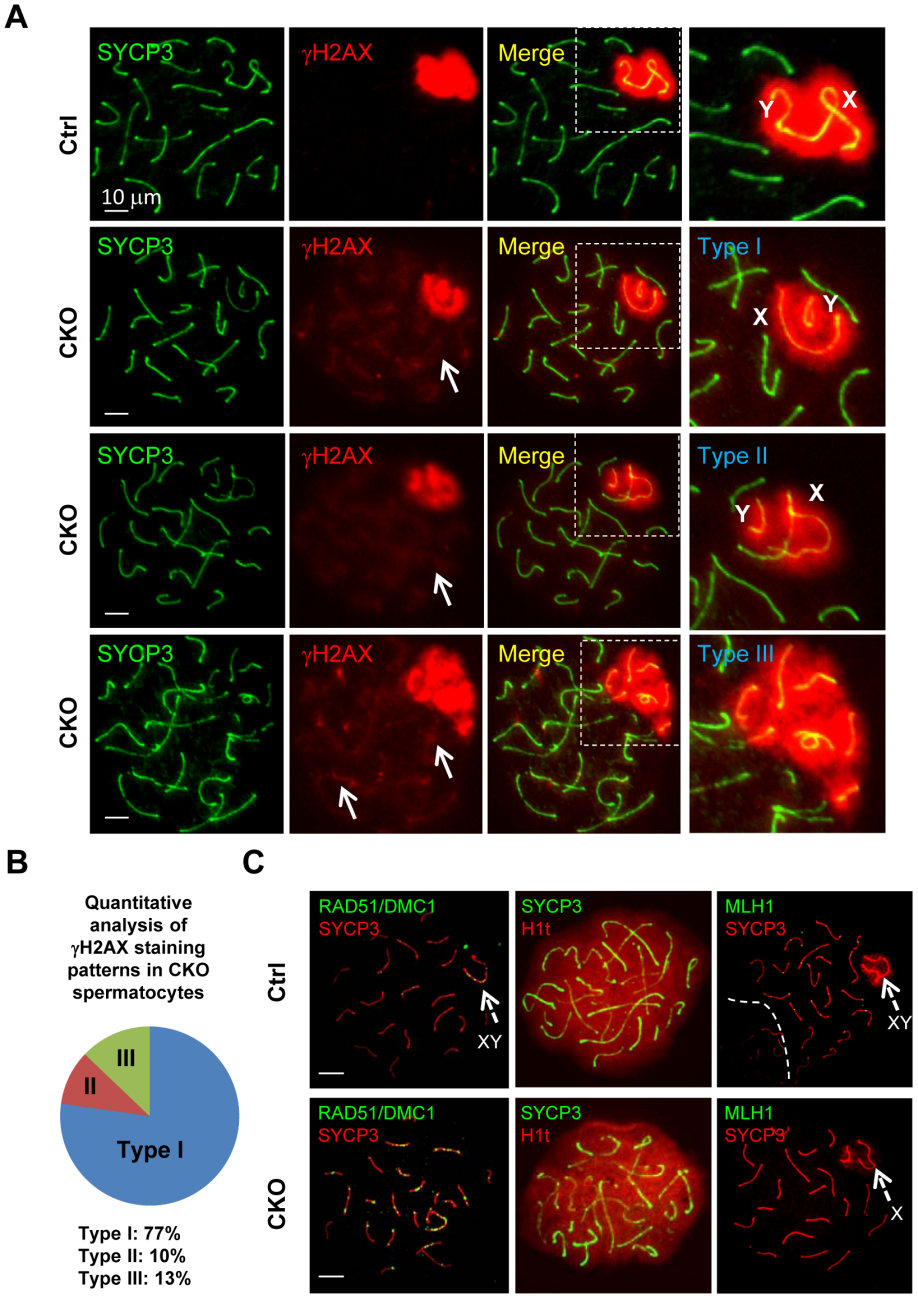
*Ikbkap* is required for XY pairing and DSB repair. (A) Representative images of immunostaining of meiotic chromosome spreads of control and CKO pachytene spermatocytes co-stained by anti-SYCP3 (green) and anti-γH2AX (red) antibodies. Areas surrounding sex chromosome are highlighted in dotted rectangles and are magnified in the right panels. Arrows indicate weak γH2AX signals on autosome. CKO spermatocytes exhibit XY synapsis defects, which were classified into three types. Type I: wide separation of X and Y chromosomes. Type II: illegitimate association of the X chromosome to autosomes. Type III: ectopic distribution of γH2AX signals on part of autosomes. Bar, 10 µm. (B) Distribution of the three types of XY synapsis defects in CKO testes (*n* = 302 nuclei, three animals for each genotype were analyzed). (C) Representative images of chromosome spreads from control and CKO spermatocytes co-stained with, RAD51 (green) and SYCP3 (red) antibodies or SYCP3 (green) and H1t (red) antibodies, or MLH1 (green) and SYCP3 (red) antibodies. Bar, 10 µm. Arrows indicate sex chromosome.

To further characterize the XY synapsis defect, we stained spermatocytes for γH2AX (a phosphorylated form of histone H2AX), a marker of DSBs, which are abundant in the silenced sex body. At early stage of prophase I, phosphorylation of H2AX is induced by SPO11-catalyzed DSBs in meiotic DNA [5]. γH2AX exhibits a diffuse staining pattern during the leptotene and zygotene stages, and becomes exclusively localized on the sex chromosomes (so called sex body) within pachytene and diplotene spermatocytes [29], [30]. We observed slightly decrease of γH2AX staining in CKO leptotene and zygotene spermatocytes as compared to the control, indicating inefficient initiation of DSB in the CKO testes (Figure S4). At pachytene stage, γH2AX localization was only restricted to the sex body, but not autosomes in the control spermatocytes (Figure 4A). Although sex bodies are formed in the CKO pachytene spermatocytes, the γH2AX signals were more concentrated in chromatin surrounding the XY axes rather than in more distant region of chromatin (Figure 4A). Moreover, not only weak γH2AX foci were persistent abnormally in the pachytene stage of CKO chromosome (Figure 4A, arrows), but more than one localized γH2AX signals were frequently observed in CKO spermatocytes (Figure S5). We further confirmed that the axes of the autosomes in CKO spermatocytes were indeed covered by γH2AX cloud using confocal microscopy (Figure S5). These results suggest an accumulation of unrepaired DSBs. Next, we analyzed sex chromosome-specific synaptic defects in details, and found that 77% of mutant spermatocytes exhibited distinct dissociation of X and Y axis (Type I in Figure 4A), whereas 10% represented illegitimate association of an end of X axis to autosomes (Type II in Figure 4A), and 13% displayed persistent γH2AX signals along the autosomes multiple chromosome within sex body (Type III in Figure 4A) (distribution of the three types is presented in Figure 4B). Taken together, our results indicate that *Ikbkap* is essential for both autosomal and XY synapsis as well as DSB repair.

The persistence of γH2AX foci in CKO spermatocytes prompted us to investigate a possible DSB repair defect. In normal meiotic recombination, SPO11-induced DSBs are repaired by the eukaryotic RecA homologs RAD51 and DMC1 (meiosis specific), which catalyze the invasion and strand exchange reaction between non-sister chromatids on homologous chromosomes [31]. To this end, we characterized the distribution of the RAD51 and DMC1 recombinases by immunostaining with an anti-RAD51 antibody that recognizes both proteins. In normal meiosis, RAD51 formed numerous foci at leptotene and zygotene stage, but these foci disappeared from autosomal axes and remained only at unsynapsed region like X and Y axis during pachytene stage [32], [33]. Consistent with a decreased γH2AX staining pattern in the CKO spermatocytes, the number of RAD51/DMC1 foci was significantly decreased in the CKO leptotene and zygotene spermatocytes as compared to the control (Figure S6). These results indicate a defect in early meiotic recombination. Moreover, while RAD51/DMC1 foci were detected in sex chromosome of the control pachytene spermatocytes (Figure 4D), they remained not only on the X axis, but also in autosomes in CKO pachytene spermatocytes. These results suggest an impairment in DSB repair in *Ikbkap* mutant spermatocytes. We next asked whether meiotic process can past the mid-pachytene stage in CKO spermatocytes by staining for Histone 1t (H1t), a mid-pachytene marker. Both control and the remaining CKO spermatocytes at mid/late pachytene stage expressed H1t (Figure 4C), suggesting that the remaining CKO spermatocytes progressed to mid-pachytene stage. To examine whether the defect in early recombination events led to the development of reciprocal exchanges (crossovers) between homologous chromosomes, we examined the distribution of mismatch repair protein MLH1, which marks the locations of crossovers [34]. Mid-pachytene control spermatocytes had 1–2 MLH1 foci on each synapsed chromosome and ∼23 per nucleus (Figure 4C). In contrast, a decreased number of MLH1 foci per nucleus were observed in CKO spermatocytes (Figure 4C), suggesting a defect in crossover formation in CKO spermatocytes.

### *Ikbkap* deletion results in aberrant transcription

**Figure 5:**
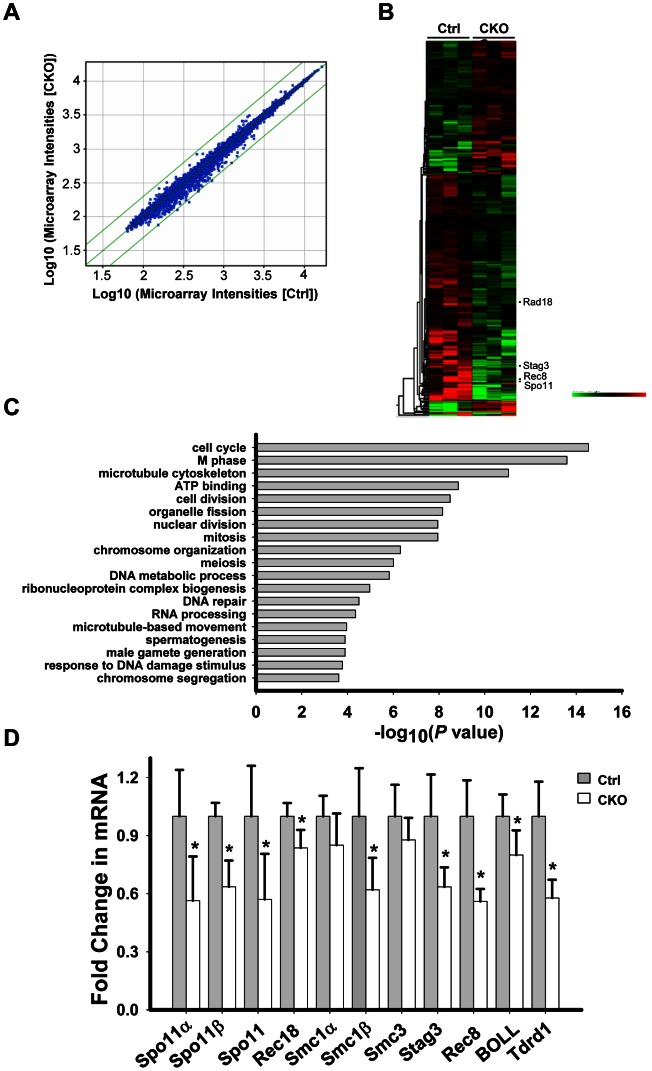
*Ikbkap* deletion results in transcriptional alteration. (A) Scatter plot of the microarray data in control and CKO testes at P15. Green lines represent differences of 2-fold from the center line. The plot is on the log scale. (B) Heatmap illustration of the genes with significantly changed (*P*<0.05). (C) Gene ontology (GO) analysis for the significantly changed genes identified in (A). A representative selection of significant GO categories is shown. (D) RT-qPCR verification of the selected genes using RNAs isolated from P15 control and CKO testes. N = 4. (*) *P*<0.01.

We next sought to investigate the underlying mechanism by which *Ikbkap* deficiency causes spermatogenic arrest in pachytene stage. Depletion of IKAP in human cells has been previously liked to transcriptional and cell migration defects [35]. To identify potential *Ikbkap*-regulated genes in meiosis, we performed microarray analysis using RNAs purified from control and CKO P15 testis. We chose to use P15 testis because the first wave of spermatogenesis progresses to the pachytene stage around this time. We observed that the levels of 1810 transcripts were significantly altered (paired t-test, *P*<0.05). However, the changes are all less than 2-fold, as indicated by scatter plot analysis (Figure 5A). Among the altered transcripts, 1103 were down-regulated, as illustrated by the heat map (Figure 5B). Gene ontology (GO) analysis revealed that the affected genes that are most enriched are involved in cell cycle and M phase processes (*P* value = 10^−14^∼10^−15^). Other terms with a significant *P* value (<10^−4^) include meiosis, DNA repair, spermatogenesis and male gamete formation (Figure 5C). By comparing *Ikbkap*-affected genes with a list of genes that were previously demonstrated to be required for synapsis, we identified *Spo11*, a type II like topoisomerase (including α and β isoforms), *Rad18* (ubiquitin ligase), and subunits of cohesion, including *Smc1β*, *Rec8* and *Stag3*. RT-qPCR analysis confirmed their down-regulation in CKO testes (Figure 5D). In addition, we verified the down-regulation of several spermatogenesis relevant genes including the boule-like (*BOLL*), and Tudor domain containing 1(*Tdrd1*) (Figure 5D). Interestingly, *Spo11*, *Smc1β, Rec8* and *Rad18* are known to play a role in meiotic DSBs repair. Given the phenotypical similarity between *Ikbkap*, *Spo11* [36], [37], *Smc1β* [38], *Rec8* [39], [40] and *Rad18* mutants [41], we believe that down-regulation of *Spo11*, *Smc1β*, *Rec8* and *Rad18* at least partly contribute to the *Ikbkap* CKO phenotype.

### *Ikbkap* is indispensable for meiotic sex chromosome inactivation

**Figure 6:**
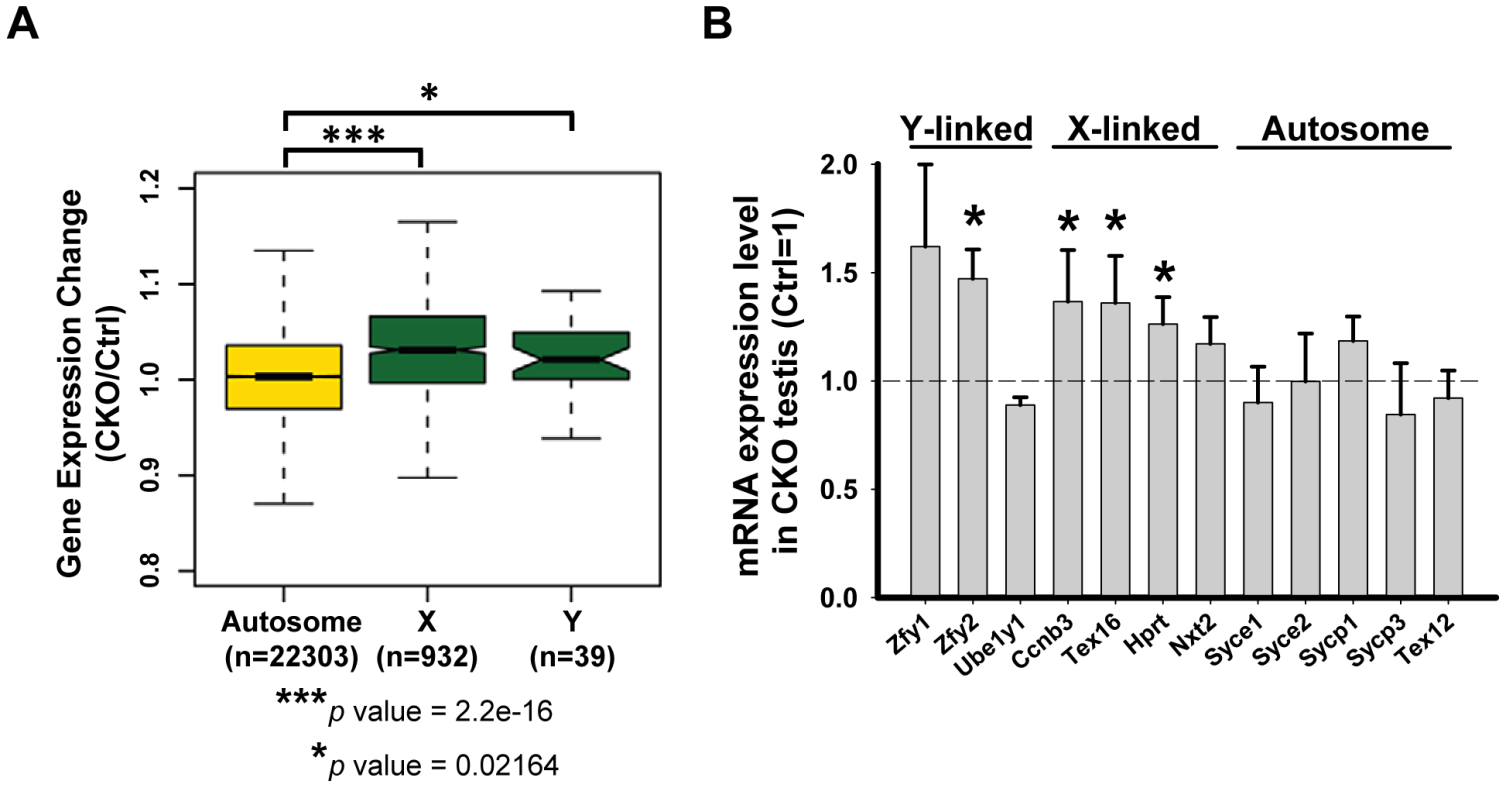
*Ikbkap* is required for MSCI. (A) Microarray analysis of control and CKO testes at P15. Comparison of gene expression levels in control and CKO testes among autosomes or X and Y chromosomes. (***) *P*<0.001. (*) *P*<0.01. (B) RT-qPCR verification of the selected genes on sex chromosomes or autosomes using RNAs isolated from P15 control and CKO testes. N = 4. (*) *P*<0.01.

In mammals, heterologous unsynapsed chromatins (sex chromosomes) are transcriptionally silenced during meiosis, a phenomenon called “meiotic sex chromosome inactivation” (MSCI) [42]. Failure in MSCI leads to apoptosis of pachytene spermatocytes, which has been proposed to be the reason for the elimination of the spermatocytes of asynaptic mutants, such as *Spo11* or *Dmc1* [43], [44]. Given the sex chromosome synapsis defect exhibited in the *Ikbkap* mutant, we asked whether it affects MSCI. At P15, when MSCI is established in normal meiosis, genes on the sex chromosomes were not repressed and were significantly up-regulated in CKO testes as compared with control testes (Kolmogorov-Smirnov test, *P*<0.05) (Figure 6A). We furthered confirmed the sex chromosome specific up-regulation in CKO testes as compared to autosomes (Figure 6A). To further validate the results, we analyzed the gene expression level of selected X-, Y-, and autosome-linked genes by RT-qPCR. Among the three Y-linked genes (*Zfy1*, *Zfy2*, *Ube1y1*) analyzed, *Zfy2* was significantly up-regulated in CKO testes (Figure 6B). We also analyzed four X-linked genes that are expressed in meiotic and postmeiotic cells (*Ccnb3*, *Nxt2*), or in premeiotic cells but repressed in meiotic and postmeiotic cells (*Tex16*, *Hprt*) [45]. In addition to few autosomal genes that we examined in Figure 5D, we also examined additional five autosomal genes that are expressed in meiotic cells (*Syce1*, *Syce2*, *Sycp1*, *Sycp3* and *Tex12*). While the expression of the autosomal genes was either not altered or down-regulated, the X-linked genes *Ccnb3*, *Tex16*, and *Hprt* showed significant increase in CKO testes (Figure 6B). Taken together, only X- and Y-linked genes, which are normally repressed during prophase I in male germline, showed increased expression in CKO testes, suggesting that *Ikbkap* is important for MSCI.

### Mammalian *Ikbkap* is required for synthesis of mcm^5^ and ncn^5^ side chains at wobble uridines

**Figure 7:**
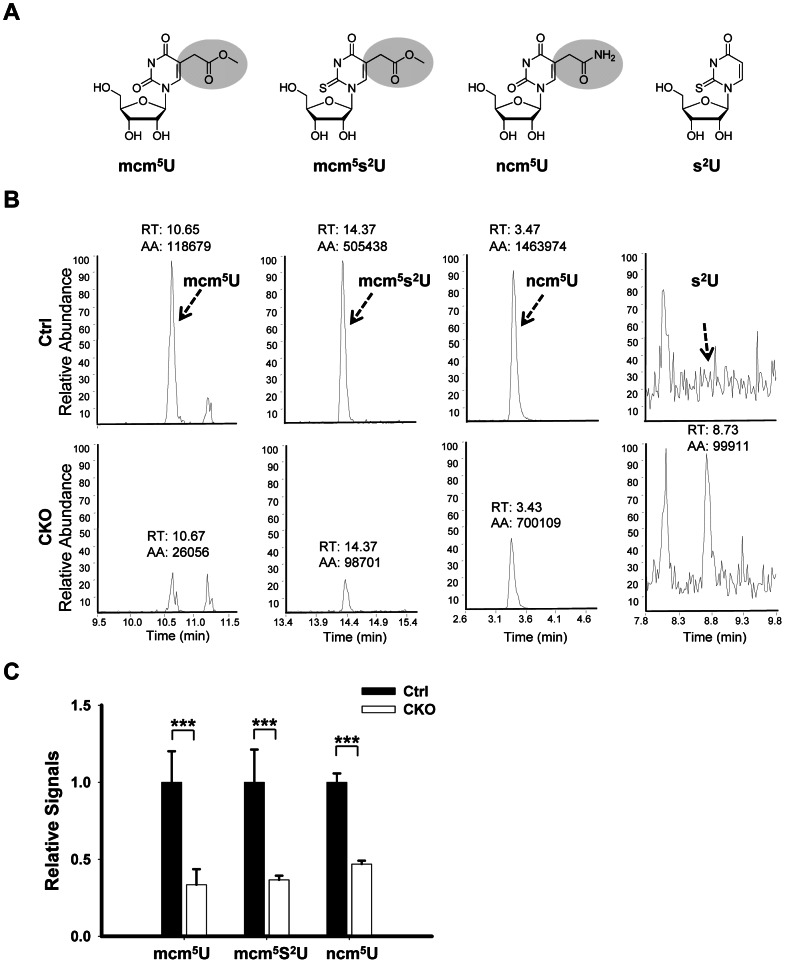
*Ikbkap* is required for the formation of mcm^5^U, ncm^5^U, and mcm^5^s^2^U in tRNA. (A) Chemical structure of the nucleosides investigated in this study. (B) LC-MS-MS chromatograms for mcm^5^U, mcm^5^s^2^U, ncm^5^U, and s^2^U of tRNAs purified from control and CKO testes at P15. The arrows indicate the signal of the modified nucleosides and the expected retention time of s^2^U. The parts of the chromatograms between the indicated retention times are displayed. (C) The levels of mcm^5^U, mcm^5^s^2^U and ncm^5^U were quantified by integration of the normalized peak intensity for each nucleoside signal and were normalized with the levels of total uridines (U). N = 4. (***) *P*<0.001.

Elongator has been shown to directly interact with tRNA *in vitro* [11], [18] and is required for wobble uridine tRNA modification in yeast, plant and worm [18], [22], [23], [24].To determine whether this function is conserved in mouse, we asked whether wobble uridine tRNA modification is affected by *Ikbkap* deletion. To test this possibility, total tRNA was extracted from P15 testis and subjected to nucleotide digestion before LC-MS-MS analysis. We used synthetic nucleoside standards to determine the retention time and nucleoside-to-base ion transition. Similar to *S. cerevisiae*, *S. pombe* and *C. elegans* tRNAs, and in accordance with previous studies [46], we found that mouse tRNA contain mcm^5^U, ncm^5^U, and mcm^5^s^2^U (Figure 7A and 7B). Importantly, the levels of mcm^5^U, ncm^5^U, and mcm^5^s^2^U in tRNA are significantly reduced in the CKO testes (Figure 7B). Quantification indicates that mcm^5^U, mcm^5^s^2^U, and ncm^5^U levels in the tRNAs of the CKO testes are only about 33%, 37%, and 47% that of the control levels, respectively (Figure 7C). Similar results were obtained from tRNAs isolated from P21 or 2 month-old testes (data not shown). tRNA from Elongator mutants of budding yeasts showed accumulation of 2-thio uridine (s^2^U), which is absent in wild-type tRNA, probably reflecting the thiolation of unmodified wobble uridine [18]. Indeed, we found correspondingly that, while s^2^U was readily detectable in the tRNAs of the CKO testes, it was not detectable in control testes (Figure 7B). This result suggests that IKAP deficiency causes accumulation of unmodified wobble uridine, some of which is thiolated into s^2^U. Taken together, our result suggests that, similar to yeast Elp1p and *C. elegans* ELPC-1, mouse IKAP is responsible for early steps of mcm^5^s^2^U, and ncm^5^U modification of tRNAs. The incomplete elimination of the formation of wobble uridine modification in CKO testes could be due to the incomplete deletion of *Ikbkap* and/or possible compensation by other pathways.

## Discussion

Elongator has been shown to play important roles in both transcription and translation, and is regarded as an essential regulator of normal development. Indeed, germ-line deletion of *Ikbkap* in mouse causes embryonic lethality at E10 likely due to impaired cardiovascular development and/or function [25], [26]. Loss of function of any of the six subunits of the Elongator impairs development in a wide variety of organisms, including yeast, fly and worm. In this study, we demonstrate that loss of function of IKAP results in multiple meiotic defects including defects in synapsis, DSB repair, and meiotic progression. In addition, we show loss of function of IKAP results in down-regulation of meiotic genes. Furthermore, we show that IKAP is required for the formation of mcm^5^ and ncm^5^ at U_34_ in tRNA. Taken together, our results support the notion that Elongator is required for tRNA modification and this function is conserved in all eukaryotes.

### *Ikbkap* is required for synapsis and DSB repair during meiosis

Key events in meiotic prophase I include: (1) introduction of SPO11-dependent double-stranded breaks (DSBs), (2) synapsing of the homologous chromosomes, (3) meiotic sex chromosome inactivation (MSCI), and (4) repair of DSBs by homologous recombination [2]. *Ikbkap*- deficient spermatocytes arrest at pachytene stage and show various meiotic phenotypes, including aberrant homologous and sex chromosomal synapsis, accumulation of unrepaired DSBs, lack of crossing over, as well as defective MSCI. Defects in synapsis and DSB repair are observed in CKO spermatocytes, suggesting that *Ikbkap* plays a role in these processes. One of the possible causes of meiotic arrest in the CKO spermatocytes could be activation of a pachytene checkpoint. Spermatocytes with defects in chromosome synapsis and/or recombination commonly trigger pachytene checkpoint control that can delay or arrest meiosis at the pachytene stage of prophase I [47]. However, the accumulation of mid-pachytene marker H1t suggests that the remaining CKO spermatocytes transits past mid-pachytene stage, which is later than pachytene checkpoint (Figure 4C). Another plausible explanation for spermatocyte elimination could be defective MSCI. MSCI is a quality control system unique to spermatocytes, and malfunction of MSCI is sufficient to trigger apoptosis of the pachytene spermatocytes [48], [49]. Indeed, we observed up-regulation of transcripts from the sex chromosomes in the CKO spermatocytes, suggesting a deficiency in MSCI. In particular, *Zfy2* expression was significantly up-regulated (Figure 6B). *Zfy1/2* paralogs are thought to be stage IV killer genes as ectopic expression of *Zfy1/2* in XY males is sufficient to phenocopy the pachytene arrest phenotype [43]. Such spermatocytes undergo apoptosis and are eliminated at stage IV of the testicular epithelial cycle. Taken together, our results suggest that loss of *Ikbkap* in germ cells likely triggers a pachytene checkpoint, which together with defective MSCI leads to spermatocyte arrest and apoptosis.

### *Ikbkap* deficiency results in aberrant meiotic gene expression

Another question raised in our study was how Elongator contributes to meiotic defects in male germ cells. Our study showed that the expression of major meiotic genes involved in synapsis, including *Spo11* (inclusive of α and β isoforms), *Rad18*, *Smc1β*, *Rec8* and *Stag3* are down-regulated in P15 juvenile testes. Among them, *Smc1β*, *Rec8* and *Stag3* belongs to the cohesin complex which provides sister chromatid cohesion and ensures chromosome segregation in mitosis and meiosis [50]. In mammalian germ cells, meiotic-specific cohesin complex contains four evolutionarily conserved protein subunits: two SMC (structural maintenance of chromosomes) proteins, SMC1β and SMC3, which heterodimerize, and two non-SMC subunits, REC8 and STAG3 [50]. They form a ring-shaped structure which embraces sister chromatids [51]. Knockout mouse models for SMC1β [38] and REC8 [39], [40] have been developed. While male meiosis of *Smc1β*-deficient mice is blocked in pachytene stage, *Rec8*-deficient spermatocytes could not proceed to pachytene. Interestingly, both *Smc1β* and *Rec8*-deficient mice show severe defects in synapsis, recombination, as well as crossover [38], [39], [40], which phenocopy *Ikbkap* meiotic phenotypes. In addition to cohesion complex, SPO11, which introduces DSBs during meiotic prophase, was also down-regulated in CKO spermatocytes. Given that *Spo11* deficiency results in failure in the initiation of meiotic recombination [36], [37], inefficient generation of DSBs in CKO leptotene/zygotene spermatocytes might result from the down-regulation of *Spo11*. Furthermore, *Spo11α*, one of major *Spo11* isoforms, and *Rad18*, are important for XY pairing. In contrast to high expression of *Spo11β* in the early prophase, *Spo11α*, a smaller isoform of *Spo11*, is highly expressed in mid- to late prophase [52]. Importantly, the XY pairing takes place later in meiotic prophase than autosomal pairing. In fact, mice that lack *Spo11α* exhibit abnormal synapsis in sex chromosomes while autosomal homologous pairing and synapsis are normal, suggesting that *Spo11α* plays a role in XY synapsis [53]. RAD18, an E3 ubiquitin ligase, has an essential function in the repair of meiotic DSBs and loss of function of RAD18 also results in XY asynapsis [41]. Therefore, it is likely that down-regulation of *Smc1β*, *Rec8*, *Stag3*, *Spo11*, and *Rec18* is at least partly responsible for the CKO phenotype. Whether *Ikbkap* directly regulates these genes in the male germ cells by facilitating their transcriptional elongation remains to be determined. Despite a predominant cytoplasmic location, a few studies have reported that in certain organisms, some subunits of the Elongator can localize to the nucleus [14], [15], [54], [55], [56], [57]. Moreover, studies in human cells have demonstrated that Elongator is preferentially recruited to the open reading frames of a number of genes [35], supporting a role for *Ikbkap* in transcription. Our results also suggest that *Ikbkap* positively regulates critical genes involved in synapsis and autosomal DSB repair. Due to the lack of chromatin immunoprecipitation (ChIP)-grade IKAP antibodies, we were unable to address whether IKAP directly contributes to transcription regulation by performing ChIP assays. Thus, we cannot exclude the possibility that IKAP contributes to transcription indirectly.

### Mammalian Elongator is involved in wobble uridine tRNA modification

Accumulating evidence indicate that Elongator has an important role in tRNA modification, which has been well-documented in several model systems, including yeasts, nematode, and plants [18], [22], [23], [24]. However, it remains unknown whether this function of the Elongator is conserved to mammals. In this study, we present the first evidence demonstrating that Elongator complex is required for the formation of mcm^5^ and ncm^5^ side chains at wobble uridines of tRNA in mammalian cells, supporting its conserved function in all eukaryotes. The conservation of this function raises the question of whether the Elongator complex itself is directly involved in the wobble uridine tRNA modification. One previous study has shown that the S-adenosyl-methionion (SAM) binding domain present in Elp3 is able to transfer methyl groups to RNAs [58]. In addition, mutations in the conserved residues of the histone acetyl transferase (HAT) domains of Elp3 also affect tRNA-modifying activity [18]. Therefore, Elp3 appears to harbor at least two enzymatic activities. Interestingly, recent studies on the crystal structure of the Elp4–6 sub-complex have revealed its potential role in substrate recognition and tRNA modification [11], [59]. Elp4, Elp5, and Elp6 all share the same RecA-like protein fold, and Elp4/5/6 forms a hetero-hexameric conformation resembling hexameric RecA-like ATPase [11]. The ring-like structure of the sub-complex together with the hydrolysis of ATP are essential for its binding to the anti-codon stem-loops of tRNA as mutations in the homologous nucleic acid binding loop (L2) of Elp6 resulted in the loss of tRNA binding capacity [11]. Thus, it is possible that removal of IKAP may affect the integrity of the complex and thereby affecting its function in tRNA modification. This possibility is supported by studies in yeast and human cells demonstrating that deletion of *Ikbkap* leads to the loss of Elp3 as well as the integrity of the Elongator [35], [60]. Further structural and enzymological analyses of the Elp1–3 sub-complex and the Elongator holo-complex will help clarify the mechanism by which Elongator contributes to tRNA modification.

Interestingly, phenotypes observed in Elongator mutants, including those in RNAPII transcription and exocytosis, could be suppressed by overexpression of two tRNAs (tRNA^Lys^_UUU_ and tRNA^Gln^_UUG_) in budding yeast [21], suggesting that the phenotypes are caused by lack of mcm^5^s^2^U modification on certain tRNAs. Therefore, it is likely that the primary effect of *Ikbkap* deficiency in germ cells is caused by tRNA modification defect, rather than dysregulation on transcription.

Mutations in the human *Ikbkap* genes have been shown to cause familial dysautonomia (FD) (also known as Riley-Day syndrome). FD is an autosomal recessive disease characterized by defects in the development and maintenance of autonomic and sensory nervous system [61], [62]. FD has been mainly associated with a single nucleotide substitution in the splice site of intron 20 of the *Ikbkap* gene, which ultimately leads to decreased expression of IKAP in a tissue-specific manner. Dietrich *et al.* has generated mice harboring exon 20 deletion allele (*IkbkapΔ20*) which phenocopy *Ikbkap* null mutations [26]. The mutants display severe cardiovascular phenotypes and die at E10 [26]. To circumvent the embryonic lethality of *Ikbkap* mutants, they further generated *Ikbkap ^flox/flox^* mice with exon 20 floxed (referred to as *Ikbkap ^floxE20/floxE20^* hereafter). In contrast to our *Ikbkap ^flox/flox^* mice, which are viable and normal, *Ikbkap ^floxE20/floxE20^* mice display low body weight, and skeletal and neuronal abnormalities. Biochemical analyses showed that *Ikbkap floxedE20* allele results in severe reduction in expression of full-length IKAP protein [63]. IKAP expression in *Ikbkap ^floxE20/floxE20^* and *Ikbkap ^Δ20/floxE20^* brains is 10% and 5% that of wild-type mice, respectively. Interestingly, both models recapitulate the major phenotypic and neuropathological features, including optic neuropathy, seizures, ataxia, impaired development and maintenance of sensory and autonomic systems, reduced number of fungiform papillae on the tongue, gastrointestinal dysfunction as well as skeletal abnormalities [63]. Our study suggests that IKAP-mediated tRNA modification may play a role in the pathogenesis of FD. Characterization of a brain-specific *Ikbkap* knockout model may reveal how IKAP contributes to FD.

## Materials and Methods

### Animal experiments

A mouse line harboring a *FRT*-flanked βGeo cassette upstream of *loxP*-flanked exon 4 of *Ikbkap* gene was obtained from Knockout Mouse Project (KOMP) Repository. *Ikbkap ^flox/flox^* mice were generated by crossing mice carrying the *Ikbkap^β-geo-flox^* allele to *Rosa26R-FLP* mice. Vasa-Cre transgenic mice were provided by Dr. Diego H. Castrillon [27]. All mouse strains were maintained in a mixed genetic background (129/Sv×C57BL/6) and received standard rodent chow. The primer sequences used for genotyping are listed in Table S1. Experimental animals and studies were approved by the Institutional Animal Care and User Committee (IACUC) of University of North Carolina at Chapel Hill.

### Histological analysis and immunohistochemistry

Testis tissues were fixed with 4% paraformaldehyde (PFA), dehydrated, and embedded in paraffin. For histological analysis, sections (7 µm) were stained with hematoxylin and eosin (H&E) or periodic acid schiff's (PAS). For immunohistochemistry, deparaffinized sections after antigen retrieval were blocked with 5% donkey serum and a biotin-blocking system (Dako, http://www.dako.com/). The following antibodies were used: anti-IKAP (LSBio, https://www.lsbio.com/), anti-SCP1, anti-SCP3, anti-DMC1 (Abcam, http://www.abcam.com/), anti-PLZF, anti-GATA1, anti-3β-HSD (Santa Cruz Biotech, http://www.scbt.com/), anti-c-Kit (Cell Signaling Technology, http://www.cellsignal.com/), anti-γH2AX, anti-RAD51(this polyclonal antibody recognize both RAD51 and DMC1 [9]) (Millipore, http://www.millipore.com/), anti-MLH1, anti-Ki67 (BD Biosciences, http://www.bdbiosciences.com/), anti-Tra98 (Bio Academia, http://www.bioacademia.co.jp/en/), and anti-H1t (a gift from M.A. Handel, The Jackson Laboratory). Sections were washed with 0.1% Triton X-100/phosphate-buffered saline (PBST) buffer and incubated with biotinylated secondary antibodies (Jackson ImmunoResearch, http://www.jacksonimmuno.com/). Signal detection was carried out with the Avidin-Biotin Complex kit (Vector Laboratories, http://www.vectorlabs.com/) or Tyramide Signal Amplification system (TSA, Invitrogen, http://www.invitrogen.com/). Peroxidase activity was visualized with 3,3′-diaminobenzidine (DAB, Vector Laboratories). Nuclear staining was carried out with 4,6-diamidino-2-phenylindole (DAPI; Sigma-Aldrich, http://www.sigmaaldrich.com/), and sections were mounted with fluorescence mounting medium (Dako) prior to imaging. Images were captured with a Zeiss Axiophoto fluorescence microscope or a Zeiss laser-scanning confocal microscope with a spinning disk (CSU-10, Yokogawa).

### TUNEL assays

TUNEL assays were performed on paraffin-embedded tissue sections using *In Situ* Cell Death Detection Kit (Roche, http://www.rocheusa.com/), following the manufacturer's instruction.

### Meiotic surface spread analysis

Testes were removed, decapsulated and shredded by needles in PBS, and the cell suspension was filtered with 100 mm mesh to remove the debris. The suspension was incubated with equal volume of a 2× hypotonic extraction buffer (30 mM Tris-HCl pH 8; 5 mM EDTA; 1.7% sucrose; 0.5% trisodium citrate) for 7 minutes. After centrifugation, cells were suspended with 100 mM sucrose solution to release hypotonized nuclei. A drop of nuclear suspension was spread onto slides that have been dipped in fixation solution (1% paraformaldehyde; 0.15% Triton X-100; 3 mM dithiothreitol (Sigma-Aldrich)). The slides were dried slowly in a humidified chamber for overnight, washed in 0.4% Photo-Flo solution (Kodak, http://www.kodak.com/), and dry again for storage. For immunofluorescent staining, the slides were permeabilized with 0.4% Triton X100/PBS for 20 minutes, rinsed with 0.1% tween 20/PBS, blocked with 5% donkey serum for 1 hour at room temperature (RT), and then incubated with primary antibodies at an optimized concentration overnight at 4°C. After wash, the slides were incubated with Alexa fluorophore conjugated secondary antibodies (Invitrogen) for 1 hour at RT. The standard protocol was followed as described above.

### Meiotic staging

The stage of prophase I for each spermatocyte was determined by chromosomal morphology and sex body status. We use SYCP3 and γH2AX staining to visualize the chromosomal changes and XY body, respectively.

### Microarray analysis

P15 juvenile testes from control or CKO mice (n = 3 for each genotype) were collected and total RNAs were extracted from mouse testes using Trizol (Invitrogen) and were cleanup using RNeasy kit (Qiagen). Samples were submitted to the UNC Functional Genomics Core Facility for RNA labeling, amplification, hybridization, and scanning. Samples were applied on Affymetrix Gene 1.0 ST assays (Affymetrix), and the procedures were followed according to the manufacturer's instructions. Data were analyzed and the expression patterns were presented as a scatter plot using GeneSpring GX software (Agilent Technologies).

### RT–qPCR assay

Total RNAs were extracted from mouse testes using Trizol (Invitrogen) and were cleanup using RNeasy kit (Qiagen). The RNAs were treated with DNase I and first-strand cDNA were synthesized by SuperScriptIII reverse transcriptase using random hexanucleotide primers according to the manufacturer's instructions (Invitrogen). Quantitative RT-PCR analyses were carried out using the ViiA7 Real-Time PCR System (Applied Biosystems) and FastStart Universal SYBR Green Master (Roche Applied Science). All expression data were normalized to *Gapdh*. The primer sequences for RT-qPCR are listed in Table S1.

### Mass spectrometric experiment

Mass spectrometric analysis of nucleosides was performed essentially as previously described [46], [64]. For sample preparation, 1 µg of total tRNA were heat-denatured, hydrolyzed with 90 U of Nuclease S1 (Sigma) in Buffer 1 (0.5 mM ZnSO_4_, 14 mM sodium acetate, pH 5.2) at 37°C for 1 hour (total volume is 44.5 µL), followed by the addition of 5 µL 10× Buffer 2 (560 mM Tris-Cl, 30 mM NaCl, 10 mM MgCl_2_, pH 8.3), 0.5 µg of phosphodiesterase I (Worthington) and 2 U of Calf Intestinal Alkaline Phosphatase (New England Biolabs) for an additional 1 hour (final volume 50 µL). The digested DNA was then filtered with Nanosep3K (Pall Corporation), and 10 µL of filtered samples were subjected to LC-MS/MS analysis using an UPLC (Waters) coupled to a TSQ-Quantum Ultra triple-quadrupole mass analyzer (ThermoFinnigan) using heat assisted electrospray ionization (HESI) in positive mode (spray voltage of 3000 V, API temperature of 250°C, sheath gas flow rate 35 arb, AUX gas flow rate 25 arb, capillary temperature of 285°C). Liquid chromatography (LC) was performed with a 2.1×100 mm HSS T3 1.8 µm column (Waters) with gradient elution at flow rate of 200 µl/min using 0.02% acetic acid in water as mobile phase A and methanol as mobile phase B. The gradient was 0→3.5 min, 3% B, 3.5→12.5 min, 3%→16.2%B, 12.5 →13 min, 16.2%B→30%B, 13→15 min, 30%B, 15→16 min, 30%→3%B, 16→20 min, 3%B. The eluent was directed to the mass spectrometer that was running in multiple reaction monitoring (MRM) mode, monitoring the transition of m/z 317.0 to 153.0 (mcm^5^U), m/z 302.0 to 153.0 (ncm^5^U), m/z 333.0 to 169.0 (mcm^5^s^2^U), m/z 261.0 to 129.0 (2-thio-U) and m/z 245.0 to 113.0 (U) for RNA samples.

### Fertility assay

We investigated reproductive capacities of *VasaCre; Ikbkap ^flox/flox^* male mice by mating one male with two wild-type females for 3 months. Female mice were checked for vaginal plugs each morning, and the litter sizes were recorded.

### Statistical analysis

Results are presented as mean ± SEM. Statistical analysis was carried out by Student's *t* test. The statistical analysis of boxplot was carried out by Kolmogorov-Smirnov test. *P* values less than 0.05 were considered statistically significant.

## Supporting Information

Figure S1 *Ikbkap* is efficiently ablated in CKO testes. Immunofluorescence for IKAP (green) and 3β-HSD (red) in paraffin sections of control and CKO testes at P14.. Bar, 20 µm.
(TIF)

Figure S2 Generation of germ cell-specific *Ikbkap* CKO mice. (A) Diagram of the *Ikbkap* mutant allele. Black boxes with numbers refer to the exons of the *Ikbkap* gene. A β-galactosidase/neomycin (β-Geo) cassette flanked by Flp recombinase recognition sites (FRT) was placed upstream of the Exon 4. It was later removed by crossing mice carrying the *Ikbkap^β-geo-flox^* allele to *Rosa26R-FLP* mice, which express Flp recombinase, to generate *Ikbkap^flox^* mice. (B) RT-qPCR analysis of the expression of the *Ikbkap* allele in P16 CKO mouse testes. N = 3. (C) Western blot analysis of IKAP protein expression in control and CKO testes. α-tubulin was used as an internal control. Method for Western blot analysis is described in Text S1. (D) Immunofluorescence for IKAP in seminiferous tubules of control and CKO testes at P14. Nuclei were counterstained with DAPI. Bar, 20 µm.
(TIF)

Figure S3 Presence of spermatogonia, sertoli cells and leydig cells in CKO testes. (A, B) Immunofluorescence for PLZF (A), GATA1 and 3β-HSD (B) in paraffin sections of control and CKO testes at P14. Nuclei were counterstained with DAPI. Bar, 20 µm.
(TIF)

Figure S4 Inefficient generation of DSBs in *Ikbkap* CKO leptotene and zygotene spermatocytes. Representative images of chromosome spreads from control and CKO leptotene and zygotene spermatocytes stained with antibodies against SYCP3 (green) and γH2AX (red).
(TIF)

Figure S5 The axes of the chromosomes are covered by γH2AX cloud staining. Representative confocal images from different focal plane of chromosome spreads from control and CKO pachytene spermatocytes stained with antibodies against SYCP3 (green) and γH2AX (red).
(TIF)

Figure S6 Inefficient generation of RAD51/DMC1 foci in *Ikbkap* CKO leptotene and zygotene spermatocytes. Representative images of chromosome spreads from control and CKO leptotene and zygotene spermatocytes stained with antibodies against RAD51/DMC1 (green), SYCP3 (red) and CREST (white). The number of RAD51/DMC1 foci is reduced in the CKO leptotene and zygotene spermatocytes. A total of 199±48 and 125±40 foci were observed in the control leptotene and zygotene spermatocytes, respectively (n = 40 each), as compared to 127±40 and 89.4±30 foci were counted in the CKO leptotene and zygotene spermatocytes, respectively (n = 40 each).
(TIF)

Table S1 The primer sequences for RT-qPCR and genotyping.
(DOCX)

Text S1 Supporting Experimental Procedures. Western Blot Analysis.
(DOC)
