# Supplementary figures and images for: Ikbkap/Elp1 Deficiency Causes Male Infertility by Disrupting Meiotic Progression
Source: PLoS Genet. 2013 May 23;9(5):e1003516. doi: 10.1371/journal.pgen.1003516 (PMC3662645; doi:10.1371/journal.pgen.1003516)

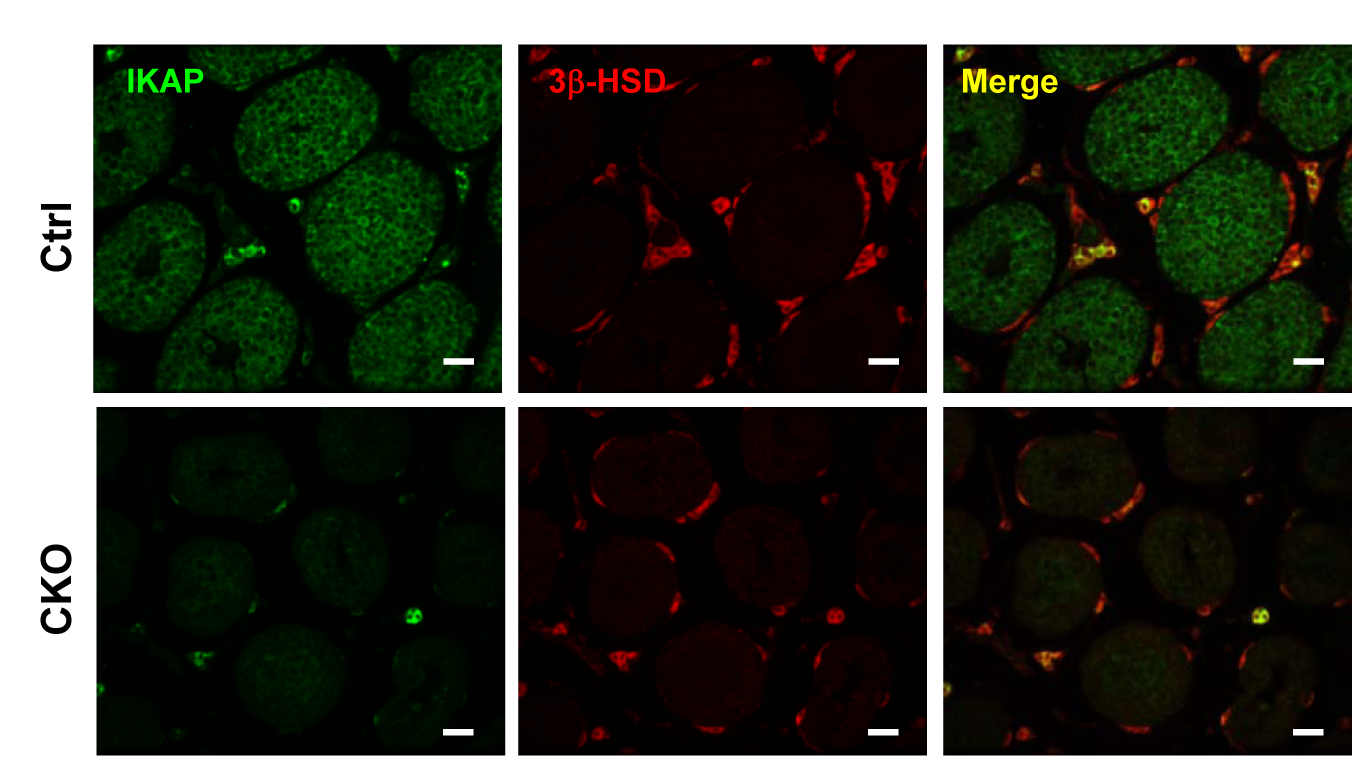

Supplement: Figure S1 — Ikbkap is efficiently ablated in CKO testes. Immunofluorescence for IKAP (green) and 3β-HSD (red) in paraffin sections of control and CKO testes at P14.. Bar, 20 µm. (TIF) [file pgen.1003516.s001.tif]

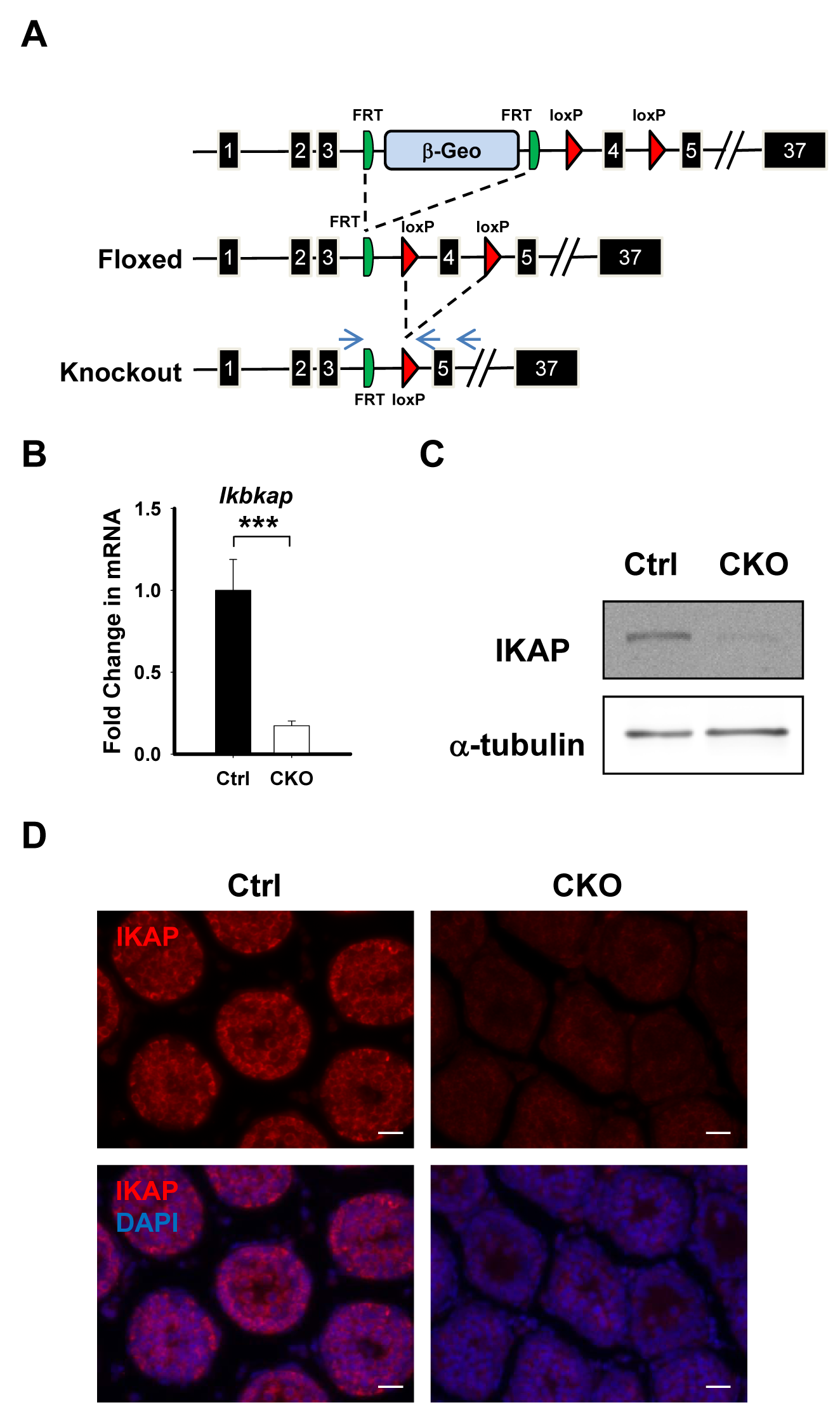

Supplement: Figure S2 — Generation of germ cell-specific Ikbkap CKO mice. (A) Diagram of the Ikbkap mutant allele. Black boxes with numbers refer to the exons of the Ikbkap gene. A β-galactosidase/neomycin (β-Geo) cassette flanked by Flp recombinase recognition sites (FRT) was placed upstream of the Exon 4. It was later removed by crossing mice carrying the Ikbkapβ-geo-flox allele to Rosa26R-FLP mice, which express Flp recombinase, to generate Ikbkapflox mice. (B) RT-qPCR analysis of the expression of the Ikbkap allele in P16 CKO mouse testes. N = 3. (C) Western blot analysis of IKAP protein expression in control and CKO testes. α-tubulin was used as an internal control. Method for Western blot analysis is described in Text S1. (D) Immunofluorescence for IKAP in seminiferous tubules of control and CKO testes at P14. Nuclei were counterstained with DAPI. Bar, 20 µm. (TIF) [file pgen.1003516.s002.tif]

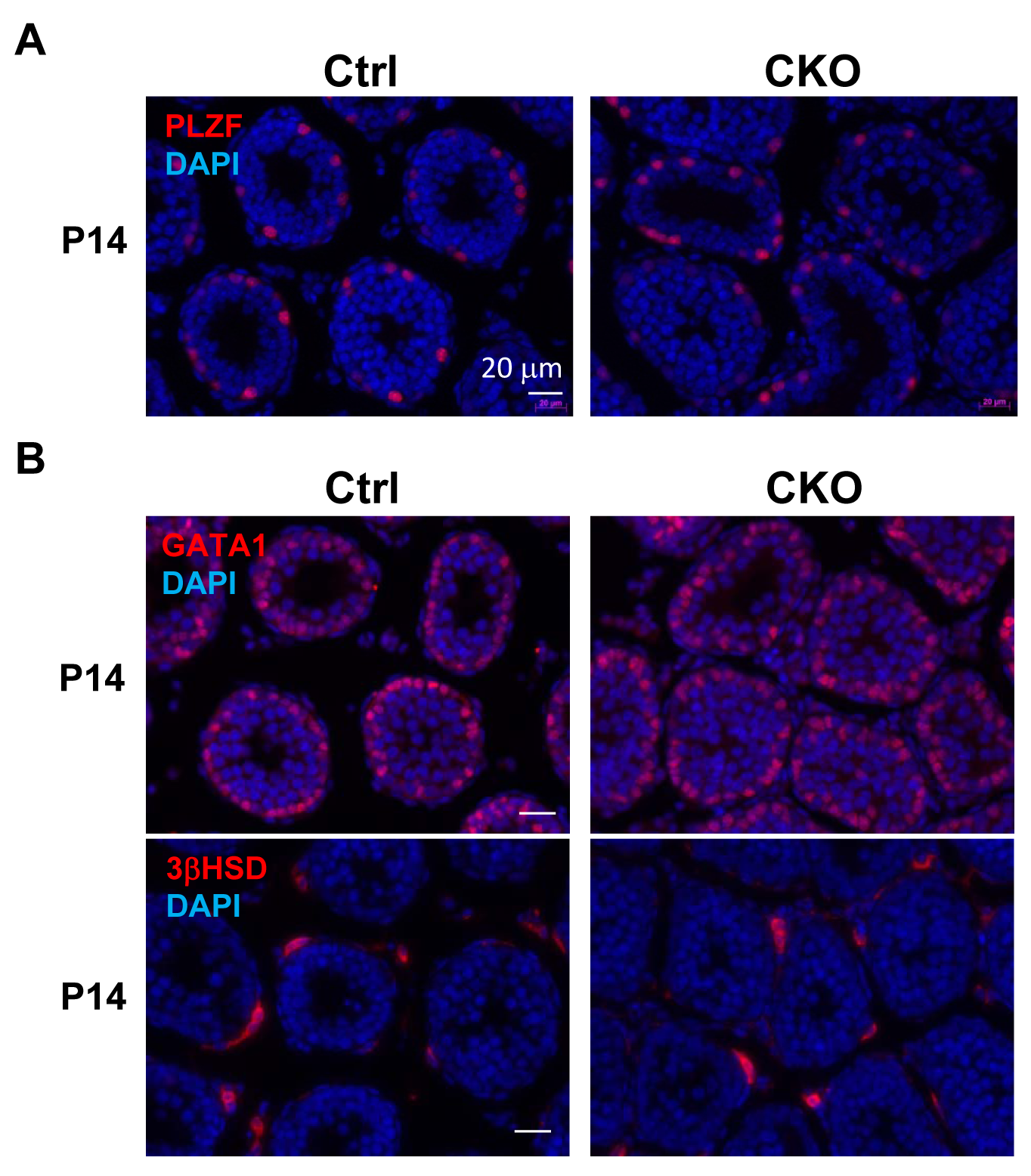

Supplement: Figure S3 — Presence of spermatogonia, sertoli cells and leydig cells in CKO testes. (A, B) Immunofluorescence for PLZF (A), GATA1 and 3β-HSD (B) in paraffin sections of control and CKO testes at P14. Nuclei were counterstained with DAPI. Bar, 20 µm. (TIF) [file pgen.1003516.s003.tif]

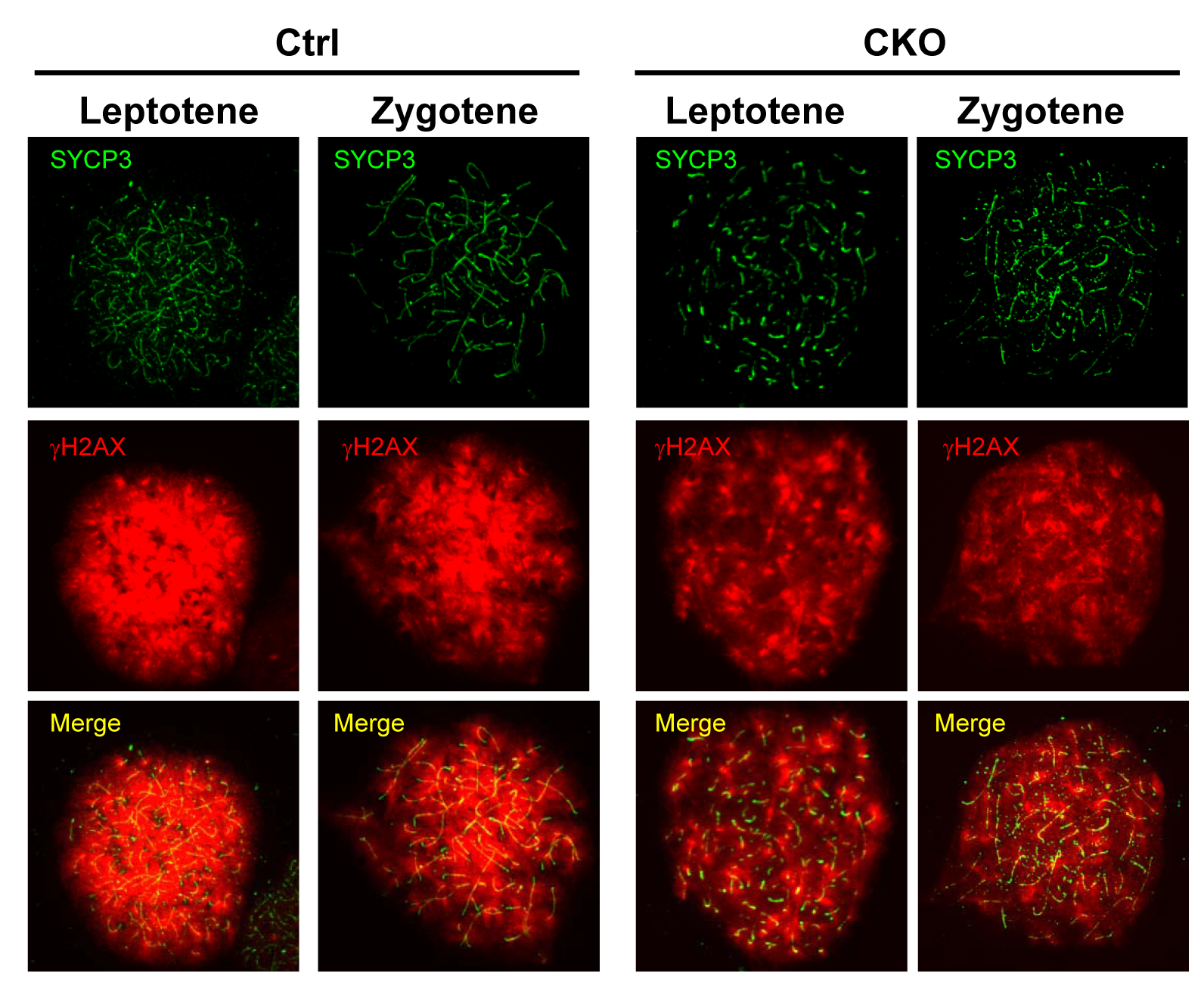

Supplement: Figure S4 — Inefficient generation of DSBs in Ikbkap CKO leptotene and zygotene spermatocytes. Representative images of chromosome spreads from control and CKO leptotene and zygotene spermatocytes stained with antibodies against SYCP3 (green) and γH2AX (red). (TIF) [file pgen.1003516.s004.tif]

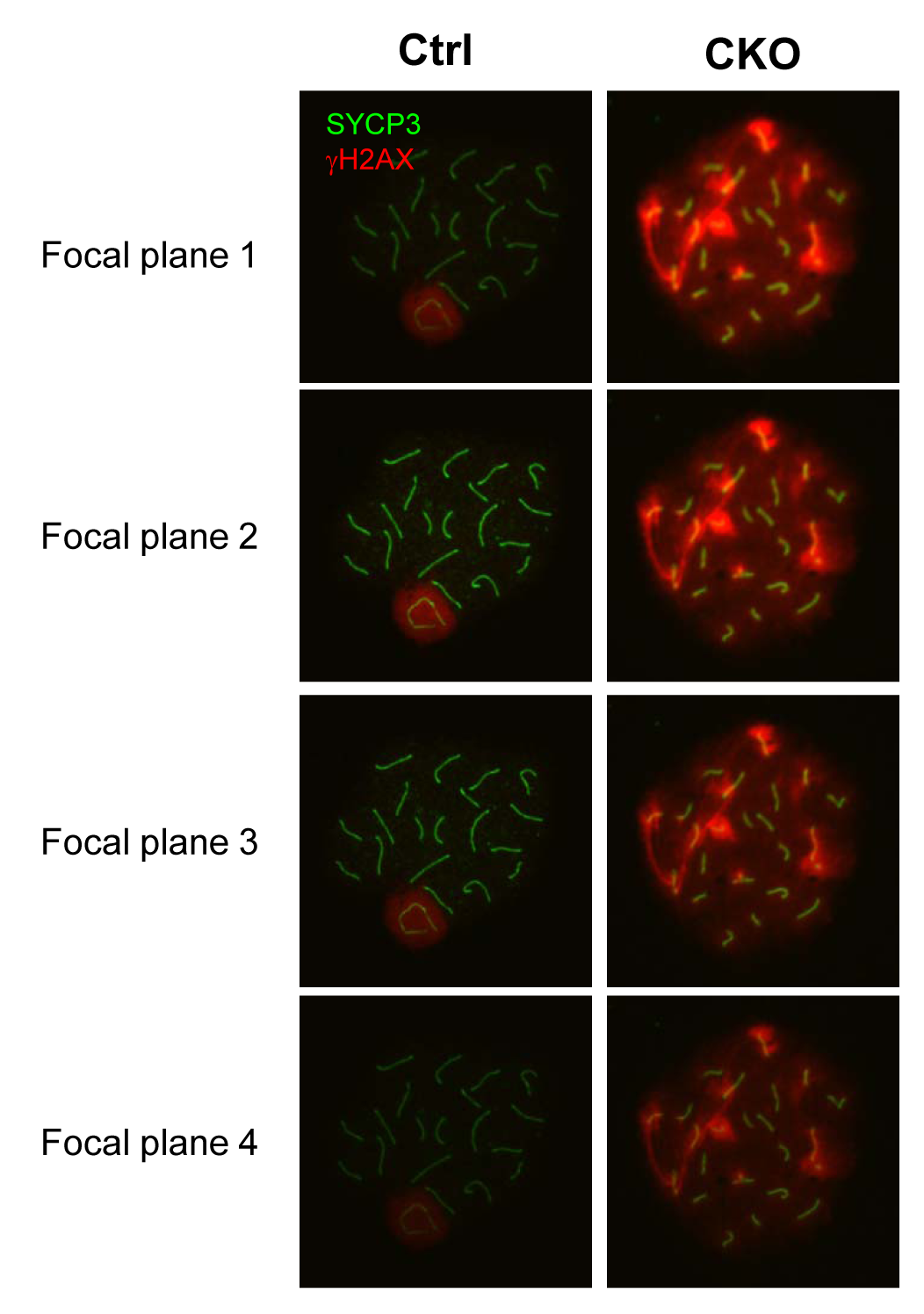

Supplement: Figure S5 — The axes of the chromosomes are covered by γH2AX cloud staining. Representative confocal images from different focal plane of chromosome spreads from control and CKO pachytene spermatocytes stained with antibodies against SYCP3 (green) and γH2AX (red). (TIF) [file pgen.1003516.s005.tif]

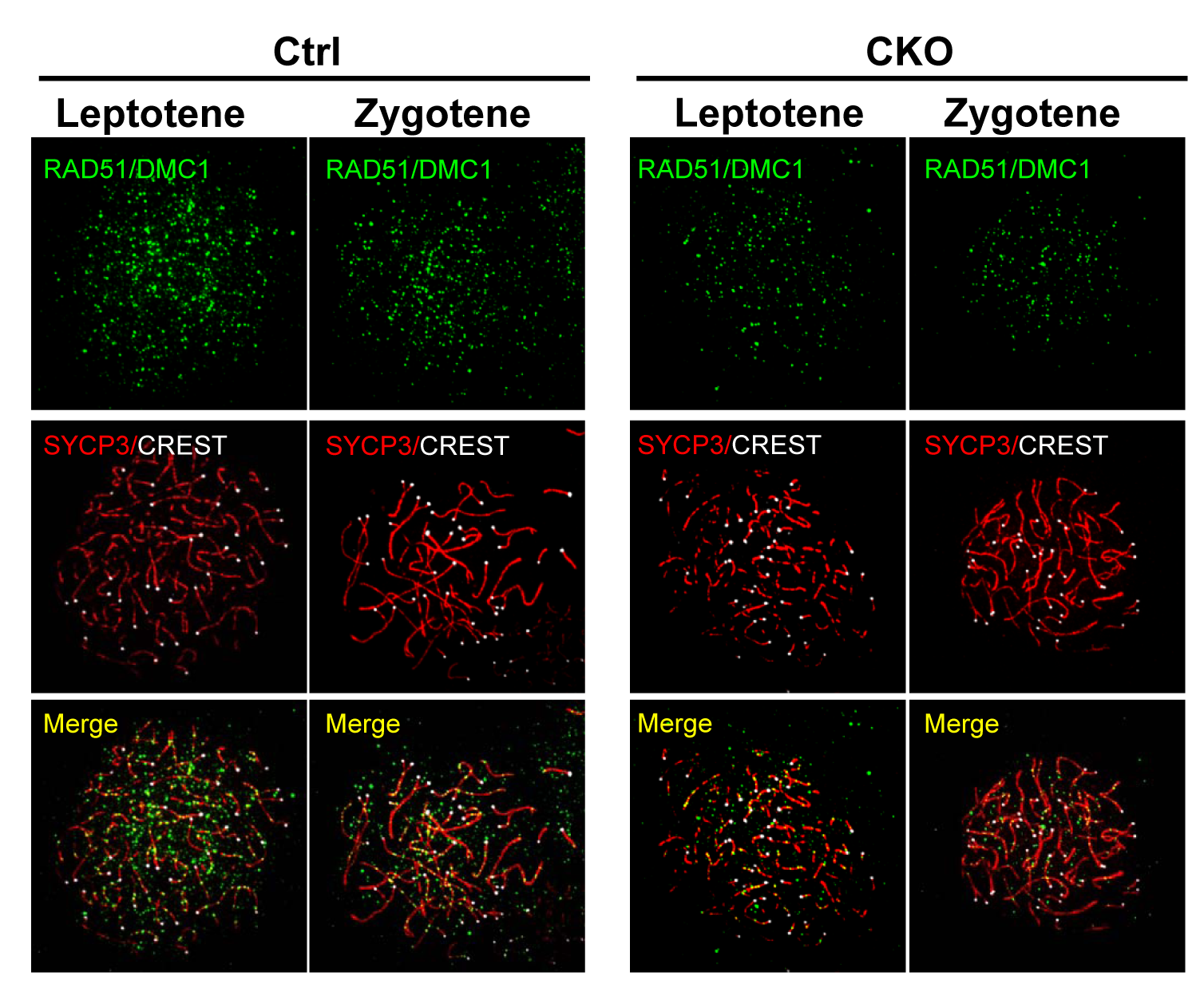

Supplement: Figure S6 — Inefficient generation of RAD51/DMC1 foci in Ikbkap CKO leptotene and zygotene spermatocytes. Representative images of chromosome spreads from control and CKO leptotene and zygotene spermatocytes stained with antibodies against RAD51/DMC1 (green), SYCP3 (red) and CREST (white). The number of RAD51/DMC1 foci is reduced in the CKO leptotene and zygotene spermatocytes. A total of 199±48 and 125±40 foci were observed in the control leptotene and zygotene spermatocytes, respectively (n = 40 each), as compared to 127±40 and 89.4±30 foci were counted in the CKO leptotene and zygotene spermatocytes, respectively (n = 40 each). (TIF) [file pgen.1003516.s006.tif]
