# Supplementary material for: Ikbkap/Elp1 Deficiency Causes Male Infertility by Disrupting Meiotic Progression
Source: PLoS Genet. 2013 May 23;9(5):e1003516. doi: 10.1371/journal.pgen.1003516 (PMC3662645; doi:10.1371/journal.pgen.1003516)
Supplement: Table S1 — The primer sequences for RT-qPCR and genotyping. (DOCX) [file pgen.1003516.s007.docx]

**Table S1.** The primer sequences for RT-qPCR and genotyping

| Gene | Primer sequences | Size (bp) | References |
| --- | --- | --- | --- |
| *Ikbkap* | F: TGCATTGTGGGTATTCAGGA R: CAGGACTCCAGCTCATGACA | 151 | This report |
| *GADPH* | F: CATGGCCTTCCGTGTTCCT R: GCGGCACGTCAGATCCA | 55 | ([Hayashi et al. 2008](#_ENREF_1)) |
| *Spo11α* | F: CGTGGCCTCTAGGTTTGATG  R: TTTTGGTGAATCGCTTCTGA | 85 | This report |
| *Spo11β* | F: GAAGTGCCTGCCTTCACAAT  R: GGCCGACAGAATCATCAAAC | 70 | This report |
| *Spo11 total* | F: TCAGATGGCTTGGTCTCCTC R: TGGTCATGCTTTGTCAGTGG | 85 | This report |
| *Smc1α* | F: AGAGTGTGCTTCAGCGGATT  R: TCGGACACTCTCCAGCTTTT | 65 | This report |
| *Smc1β* | F: TGGCCTTGTCTGTGAGACTG  R: TGCTACTGCCTTCCGTCTTT | 76 | This report |
| *Smc3* | F: TTGGAGCTTAAAGCCAAGGA  R: CAAGCAGCTTCTGCCTCTCT | 91 | This report |
| *Stag3* | F: CCTCAGGCAGTGAGTCTTCC  R: CACTCTGCAGTTCCCTGTGA | 121 | This report |
| *Rec8* | F: TTGGCTTCTCGGGTCTTCTA  R: GCCCATATGGCTTCTGTTGT | 82 | This report |
| *Rad18* | F: TGGCGGTGATGAAGACAAT R: GTGAGAGCACTGGGGGATTA | 92 | This report |
| *Zfy1* | F: GAGCGTCCTTCTCCTCACAC  R: CTGGCTGAGTGCGTCATAAA | 110 | This report |
| *Zfy2* | F: CCCTCGGAAAAGAATCAACA  R: GAATGCCAATGTCCCCATAC | 92 | This report |
| *Ube1y1* | F: GATGGGCAAGTGGCTGTATT R: CATGGCAAAGTTCTTGAGCA | 114 | This report |
| *Ccnb3* | F: CTGAGAAAGATCCCCCTTCC R: TTGGTGAGACATGCTGCTTC | 99 | This report |
| *Hprt* | F: CAGGCCAGACTTTGTTGGAT  R: TTGCGCTCATCTTAGGCTTT | 147 | This report |
| *Tex12* | F: AAACTGCAAGAGGGCAAGAG R: TCCAGAAGCCTCAGAGAGAGA | 100 | ([Yamaguchi et al. 2012](#_ENREF_3)) |
| *Tex16* | F: GCAACAATGGGTCTCAACCT R: ATTGCTGGAATCAGGTCCAC | 62 | This report |
| *Nxt2* | F: GCTCTGGAGCAGACCATTTC  R: AATTCCTCGGCAGCTCTACA | 92 | This report |
| *Syce1* | F: GAAGTCCACGAGGAGACAGC  R: TCCGCAAGTGATGTCTGAAG | 103 | This report |
| *Syce2* | F: CAAGTCTGCCAAACTGTGGA  R: GCAGAAGTCAGCATTCACCA | 106 | This report |
| *Sycp1* | F: GGACAACGATTGCTAAAATTGATAGG R: TTGGTAAAGTTTGGCTCTCTTGG | 149 | ([Yabuta et al. 2011](#_ENREF_2)) |
| *Sycp3* | F: TGAGTCTTTGAAGAAAGAACTTGAACC R: GGTTAACAGGAACAAAAATCTTCCAC | 148 | ([Yabuta et al. 2011](#_ENREF_2)) |
| Ikbkap-genotype 1 | F: CATTTGTGGCTTCTCACTGTCACAAGACA R: GAGTTCTGTGAAGGTCTCAAAGGT | 370 (floxed) 230 (WT) | This report |
| Ikbkap-genotype 2 | F: GTCGAGAAGTTCCTATTCCGAAGT R: AGCAAGGAGAGTCAAGAGGTTCTA | 350 | This report |
| Cre-genotype | F: GCTAAACATGCTTCATCGTCGG R: GATCTCCGGTATTGAAACTCCAGC | 646 | This report |

**SUPPLEMENTAL REFERENCES**

Bellani MA, Boateng KA, McLeod D, Camerini-Otero RD. 2010. The expression profile of the major mouse SPO11 isoforms indicates that SPO11beta introduces double strand breaks and suggests that SPO11alpha has an additional role in prophase in both spermatocytes and oocytes. *Mol Cell Biol* **30**: 4391-4403.

Hayashi K, Chuva de Sousa Lopes SM, Kaneda M, Tang F, Hajkova P, Lao K, O'Carroll D, Das PP, Tarakhovsky A, Miska EA et al. 2008. MicroRNA biogenesis is required for mouse primordial germ cell development and spermatogenesis. *PLoS One* **3**: e1738.

Yabuta Y, Ohta H, Abe T, Kurimoto K, Chuma S, Saitou M. 2011. TDRD5 is required for retrotransposon silencing, chromatoid body assembly, and spermiogenesis in mice. *J Cell Biol* **192**: 781-795.

Yamaguchi S, Hong K, Liu R, Shen L, Inoue A, Diep D, Zhang K, Zhang Y. 2012. Tet1 controls meiosis by regulating meiotic gene expression. *Nature* **492**: 443-447.
