# Supplementary material for: Ikbkap/Elp1 Deficiency Causes Male Infertility by Disrupting Meiotic Progression
Source: PLoS Genet. 2013 May 23;9(5):e1003516. doi: 10.1371/journal.pgen.1003516 (PMC3662645; doi:10.1371/journal.pgen.1003516)
Supplement: Text S1 — Supporting Experimental Procedures. Western Blot Analysis. (DOC) [file pgen.1003516.s008.doc]

**Text S1**

**Supporting Experimental Procedures**

**Western Blot Analysis**

Mouse testes were homogenized with 1X RIPA buffer containing 1X TBS, 1% Nonidet P-40, 0.5% sodium deoxycholate, 0.1% SDS, and a protease inhibitor cocktail (Roche) on ice. After centrifugation for 15 minutes, the supernatant was collected, and protein content of the samples was analyzed according to the Bradford method. Proteins were loaded onto SDS-polyacrylamide gels and blotted onto PVDF membranes (Bio-Rad Laboratories). Western blots were performed using antibodies directed against IKAP (Millipore or LSBio), α-tubulin (Sigma-Aldrich) Enhanced chemiluminescence was performed according to the manufacturer’s instructions (Amershan Biosciences, UK).
